# Genotype specificity among hosts, pathogens, and beneficial microbes influences the strength of symbiont‐mediated protection

**DOI:** 10.1111/evo.13216

**Published:** 2017-03-24

**Authors:** Benjamin J. Parker, Jan Hrček, Ailsa H. C. McLean, H. Charles J. Godfray

**Affiliations:** ^1^ Department of Zoology University of Oxford Oxford OX1 3PS United Kingdom; ^2^ Current Address: Department of Biology University of Rochester Rochester NY 14627 USA; ^3^ Current Address: Institute of Entomology Biology Centre CAS, Branisovska 31 Ceske Budejovice 37005 Czech Republic

**Keywords:** Coevolution, endosymbiont, fungal pathogens, mutualism, pea aphid (*Acyrthosiphon pisum*), symbiont‐mediated resistance

## Abstract

The microbial symbionts of eukaryotes influence disease resistance in many host‐parasite systems. Symbionts show substantial variation in both genotype and phenotype, but it is unclear how natural selection maintains this variation. It is also unknown whether variable symbiont genotypes show specificity with the genotypes of hosts or parasites in natural populations. Genotype by genotype interactions are a necessary condition for coevolution between interacting species. Uncovering the patterns of genetic specificity among hosts, symbionts, and parasites is therefore critical for determining the role that symbionts play in host‐parasite coevolution. Here, we show that the strength of protection conferred against a fungal pathogen by a vertically transmitted symbiont of an aphid is influenced by both host‐symbiont and symbiont‐pathogen genotype by genotype interactions. Further, we show that certain symbiont phylogenetic clades have evolved to provide stronger protection against particular pathogen genotypes. However, we found no evidence of reciprocal adaptation of co‐occurring host and symbiont lineages. Our results suggest that genetic variation among symbiont strains may be maintained by antagonistic coevolution with their host and/or their host's parasites.

Resistance to infection is determined by a host organism's own genotype and by the genotype of an infecting parasite. This is because many host‐parasite pairs are characterized by high levels of genetic specificity, where parasite genotypes (G_Parasite_) can infect only a subset of host genotypes (G_Host_), and hosts can resist only a subset of parasites (Carius et al. 2001; Schulenburg and Ewbank 2004; Auld et al. 2010). G_Host_ x G_Parasite_ specificity can lead to a process of coevolution, often referred to as Red Queen Dynamics (Van Valen 1973), where selection acts against common host and parasite genotypes (Lively and Dybdahl 2000; Lambrechts et al. 2006). This causes allele frequencies to cycle and contributes to the maintenance of genetic variation in natural populations (Woolhouse et al. 2002; Gandon et al. 2008; Koskella and Lively 2009). In many systems, resistance is also influenced by a third interacting partner—a predominately vertically transmitted microbial symbiont that protects its host from parasite infection (Haine 2008). Symbiont‐mediated protection has been documented across diverse host taxa, including plants (Arnold et al. 2003), mammals (Barton et al. 2007), and invertebrates (Teixeira et al. 2008; Oliver et al. 2014). These examples include systems where symbionts influence the vector competence of medically and agriculturally important hosts (Geiger et al. 2007; Moreira et al. 2009; Wang et al. 2009; Gottlieb et al. 2010; Sassera et al. 2013; Caragata et al. 2016; Dutra et al. 2016). Because hosts differ in the strains and species of symbionts they harbor (Ferrari and Vavre 2011), vertically transmitted microbes represent an additional source of heritable variation that can be acted on by natural selection (Jaenike 2012). What remains unclear, however, is the role genetic variation among protective symbionts plays in host‐parasite interactions, how this variation is maintained, and the degree to which current models of host‐parasite coevolution are relevant when host resistance is influenced by symbiont‐mediated protection.

Although the evolutionary dynamics of antagonistic interactions between hosts and microbes have been well studied, we know comparatively little about the dynamics of beneficial infections. Some early theoretical investigations assumed that hosts and vertically transmitted microbes both benefit by enhancing the fitness of their partners (Law and Lewis 1983). Under this scenario, it is easier for hosts to adapt to common symbiont genotypes (through positive frequency‐dependent selection), and thus selection would favor evolutionary stasis (Law and Koptur 1986; Weyl et al. 2010). However, studies have revealed high diversity within lineages of beneficial symbionts that is maintained for long periods of time (Duron and Hurst 2013; Henry et al. 2013; Martinez et al. 2014, 2015). How this variation is maintained is an important question in host‐symbiont biology (Heath and Stinchcombe 2014). Genotype by genotype interactions between protective symbionts and parasites (G_Symbiont_ x G_Parasite_) could help to explain the maintenance of genetic variation in protective symbiont lineages through the same frequency‐dependent dynamics that contribute to variation in host immune effector systems.

Symbionts also interact with variable host genotypes, either when vertically transmitted symbionts encounter novel host backgrounds produced through recombination or through horizontal transmission events. Recent molecular analyses of predominantly vertically transmitted microbes have provided evidence of relatively frequent horizontal transmission (through inter‐ and intraspecific transfers) over evolutionary time scales (Pool et al. 2006; Haselkorn et al. 2009; Raychoudhury et al. 2009; Jaenike et al. 2010; Mouton et al. 2012; Henry et al. 2013; Haselkorn and Jaenike 2015). Studies of this process have improved our understanding of how symbiont‐associated phenotypes are influenced by host genetic backgrounds. For example, symbionts often do poorly in new host environments, both because they are more costly for their hosts to carry and because they confer less beneficial phenotypes (Nakayama et al. 2015). There is also evidence of adaptation of symbionts to novel host environments—for example, *Wolbachia* that spread through a population of *Drosophila simulans* evolved to provide a fecundity advantage to infected hosts over a 20 year period (Weeks et al. 2007). In these cases, host‐symbiont coevolution may improve the efficacy of symbiont‐mediated protection, and we might expect pairings of host and symbiont genotypes that associate in natural populations to produce stronger beneficial phenotypes than nonnatural pairings. However, it is currently unclear if host and symbiont genotypes exhibit specificity (G_Host_ x G_Symbiont_) and if specificity influences the phenotypic effects of vertically transmitted microbes.

We examine the importance of genetic variation in a natural host‐symbiont‐pathogen system, that of the pea aphid (*Acyrthosiphon pisum*), the facultative symbiont *Regiella insecticola*, and the aphid‐specific fungal pathogen *Pandora neoaphidis* (Fig. 1). Pea aphids harbor several species of maternally transmitted, facultative bacterial symbionts (in addition to an obligate nutritional symbiont), a number of which have been shown to play a role in host defense (Oliver et al. 2010; Łukasik et al. 2013). *Regiella* protects aphids against *Pandora* and other specialist fungal pathogens (Scarborough et al. 2005; Parker et al. 2013), which are important natural enemies of pea aphids in wild populations (Van Veen et al. 2008). Other pea aphid symbionts have been shown to confer protection against parasitoid wasps (Oliver et al. 2003), and studies have demonstrated specificity between symbiont genotype and wasp species (Asplen et al. 2014; McLean and Godfray 2015). In addition, a recent investigation used experimental evolution to generate genetic specificity between symbiont and wasp genotypes in the laboratory (Rouchet and Vorburger 2014). These studies suggest that genotype by genotype interactions between symbionts and natural enemies may be an important force influencing evolution in natural populations. In addition, the pea aphid species comprises genetically distinct, specialized host‐plant associated populations, referred to as “biotypes” (Peccoud et al. 2009). Despite evidence of a low‐level of horizontal transmission of symbionts among aphid lineages, particular biotypes carry distinctive sets of symbiont species or genotypes (Tsuchida et al. 2002; Ferrari et al. 2012; Russell et al. 2013) suggesting the potential for coadaptation between host and symbiont lineages. We used this system to test whether (i) G_Symbiont_ x G_Parasite_ specificity and (ii) G_Host_ x G_Symbiont_ specificity influence resistance of pea aphids against fungal pathogens.

**Figure 1:**
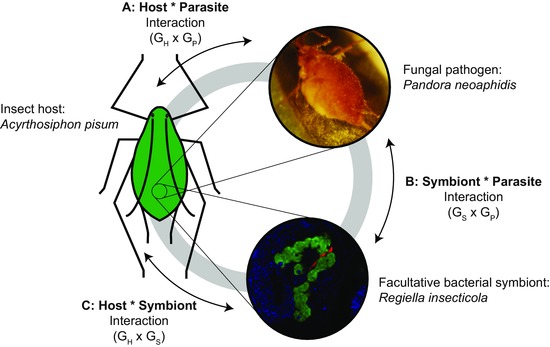
Potential 3‐way genetic interactions. (A) Genotype by genotype interactions between hosts and parasites, often mediated through host immune mechanisms, have been documented in a number of systems. The upper photo (from B. Parker) shows a “sporulating” pea aphid—an individual that has been infected with *Pandora* and is subsequently releasing spores into the environment. (B) Specific G x G interactions between symbiont and parasite genotypes may underlie symbiont‐mediated protection. The lower photo (from A. Douglas) shows specialized aphid cells (bacteriocytes and sheath cells) that house symbionts. The primary symbiont *Buchnera aphidicola* is tagged green; *Regiella* is tagged red. (C) G x G interactions between host and symbiont genotypes, potentially mediated by the host's immune system, could influence symbiont‐mediated protection.

## Methods

### APHID COLLECTION, REARING, AND GENOTYPING

The aphids and symbionts used in this experiment were primarily collected in the United Kingdom, with some symbiont strains from the United States and France (see Tables S1 and S2). We maintain aphids in the laboratory on *Vicia faba* plants at a light and temperature regime of 16L:8D and 14°C. Under long daylight regimes, aphids reproduce by apomictic parthenogenesis, which allows us to use genetically identical aphid hosts in our experiments. We used microsatellite markers to confirm that aphid clones belonged to the previously characterized biotypes associated with the plants from which they had been collected (Peccoud et al. 2009). Before use in our experiments, we screened all of the lines for the seven known pea aphid facultative symbionts. DNA was extracted using the DNeasy Blood and Tissue kit (Qiagen), and a PCR test using symbiont‐specific primers (Henry et al. 2013) was used to identify symbionts. DNA was amplified using a “touchdown” PCR (94°C 2 min, 11 cycles of (94°C 20s, 56°C (declining 1°C each cycle) 50 s, 72°C 30 s), 25 cycles of 94°C 2 min, 45°C 50 s, 72°C 2 min and a final extension of 72°C 5 min). We characterized the strains of *Regiella* used in this study using a MLST scheme developed previously (Degnan and Moran 2008; Henry et al. 2013). DNA was extracted from at least two adult aphids as above and six housekeeping genes (*accD*, *gyrB*, *hrpA*, *murE*, *recJ*, and *rpoS*) were amplified using the PCR protocol described above. We sequenced these genes in the forward and reverse direction using Sanger sequencing, and a consensus sequence was generated by aligning the two sequences in Geneious (v.7) and editing by hand. A maximum likelihood phylogeny was generated using Geneious (v.7).

### SYMBIONT CLEARING AND TRANSFER

Our protocol for clearing aphids of their facultative symbionts is based on McLean et al. (2011). We immersed the stems of *V. faba* leaves in an antibiotic solution (100 mg/mL Ampicillin, 50 mg/mL Cefotaxime and 50 mg/mL Gentomicin (Chandler et al. 2008)), and fed first‐instar aphids on the leaves for 48 hours. We then moved the aphids to fresh leaves until they became adults. We collected the late offspring of these antibiotic‐fed aphids, and when they became adults tested their offspring for symbionts using PCR. We waited a minimum of eight generations before using a cleared line in an experiment to ensure there were no maternal effects of antibiotic treatment on experimental aphids.

To transfer symbionts into uninfected lineages, we injected a small volume of hemolymph (approximately 0.25 μL) from an infected donor aphid into a one‐day‐old 1st instar recipient using a capillary needle. Injected aphids were reared until they became adults when their offspring were collected and tested for the presence of the symbiont. As above, we waited a minimum of eight generations for the symbiosis to stabilize before using a line in an experiment (Koga et al. 2003).

### *PANDORA* CULTIVATION

We obtained the *Pandora* strains used in this experiment from the USDA Agriculture Research Service collection of entomopathogenic fungi (ARSEF). Fungi in this collection were obtained from the wild, grown on artificial media, and preserved in liquid nitrogen. Before use in our experiments, isolates were thawed and grown on a modified Sabouraud dextrose agar (SDAEY) as described in Hajek and Papierok (2012). We then cut 1 cm^2^ of mycelium from these plates, and moved the fungus to tap water agar to induce sporulation. We exposed aphids to fungal spores (as in Parker et al. 2014), and placed dead infected aphids at 4°C to dry. We induced sporulation of these dried cadavers to perform subsequent infections, and repeated this procedure several times before using the isolates in the experiment. Note that we passaged fungal isolates through symbiont‐free pea aphids from an aphid genotype (Line 145) that does not belong to either the *Trifolium* spp. or *Medicago sativa* biotypes.

### FUNGAL INFECTION PROTOCOL

To perform the fungal infection experiments, we exposed aphids to sporulating conspecific cadavers (based on Ferrari et al. 2001; Scarborough et al. 2005; Parker et al. 2014). Eleven‐day‐old aphids (all recently molted to the final adult instar) were placed in the bottom of an infection chamber—a PVC tube (39 mm diameter, 55 mm height)—with sporulating cadavers placed above the chamber so spores fall onto the experimental aphids. The sides of the chamber were painted with a Teflon coating to keep the insects at the bottom of the chamber (insect‐a‐slip—Fluon, Bioquip). Sporulating cadavers were rotated among the treatment aphids so that all aphids within an experiment are exposed to each set of sporulating cadavers for an equal period of time (as in Parker et al. 2014). After fungal exposure, aphids were then transferred to Petri dishes with a leaf inserted into 2% tap water agar, with four adult aphids in each dish. The dishes were sealed with parafilm around the edges to keep the humidity high (measured at ∼100% in pilot trials), and kept at 20°C and 16L:8D for 48 hours. Aphids were transferred to a new dish, without parafilm, on the 3rd and 6th days of the infection. Each dish was assigned a number randomly so data collection was blind to treatment, and data on survival and whether an aphid sporulated were collected every 24 hours until the 8th day after infection.

### EXPERIMENTAL DESIGN AND STATISTICAL METHODS

We used these protocols to carry out the two main experiments. For the G_Symbiont_ x G_Pathogen_ experiment, we established 15 *Regiella* genotypes in a common host background, and infected these lines with three genotypes of *Pandora*. The *Regiella* genotypes used included 13 strains from pea aphids and two strains from other aphid species (see Table S2). We reared aphids for use in the experiment (only apterous morphs) on *V. faba* plants, at a density of approximately 10 adult aphids per plant. We exposed 40 aphids from each line to each strain of *Pandora*, and also included 40 unexposed aphids as a control. We scored each aphid for signs of fungal infection (the formation of a sporulating cadaver–‐Fig. 1, top right–‐and spores visible on the plant surface and Petri dish) over an 8‐day period. Data on sporulation frequencies were analyzed using generalized linear models (GLMs) with a binomial error structure, after checking for overdispersion, using R version 3.0.2 (R Core Team). Symbiont genotype was nested within symbiont clade (either “clade 1” or “clade 2” as determined by our MLST phylogeny; no clade information was included in the analysis for the two nonpea aphid outgroup *Regiella* strains). Clade was subsequently nested within symbiont presence for lines harboring *Regiella* to include symbiont‐free lines in the model. We ran all treatments within the experiment (all combinations of G_Symbiont_ and G_Pathogen_) at the same time, and therefore there were no block effects to account for in the statistical model. We performed model comparisons with ANOVA. It is important to note that although aphids within a fungal treatment were exposed to equal spore doses, we were not able precisely to control doses among different fungal genotypes, and therefore the main effect of *Pandora* genotype includes a small component due to variation in spore dose as well as genetic differences among fungal genotypes.

For the G_Host_ x G_Symbiont_ experiment, we took six pea aphid genotypes that when collected carried a natural *Regiella* infection, and generated the fully factorial set of six aphid by six *Regiella* genotypes. Note that for lines collected with coinfecting symbionts in addition to *Regiella*, we obtained single infections of *Regiella* in its native host background using antibiotic curing. As discussed above, variation in symbiont strains harbored by aphids is structured by host biotype. Specifically, *Regiella* is commonly associated with two biotypes of pea aphids: one that feeds on *Medicago sativa* and the other on *Trifolium* spp.. *Regiella* from “clade 1” are statistically significantly associated with the *Medicago* biotype while *Regiella* from “clade 2” are significantly associated with the *Trifolium* aphid biotype. However, aphids from both biotypes may be collected with strains of *Regiella* from either clade (Henry et al. 2013). In our study, we included three aphid lines each from the *Medicago* and *Trifolium* biotypes; the *Medicago* lines originally hosted *Regiella* from clade 1 and the *Trifolium* lines *Regiella* from clade 2. We looked at the effects of aphid and *Regiella* genotypes on the percentage of aphids successfully infected by *Pandora*, and for each effect further asked how much of the variation in the data is explained by grouping genotypes into host biotypes and major symbiont clades. Aphids from each line were reared and then exposed to a single genotype of *Pandora* (strain ARSEF 2588, chosen randomly from the genotypes used in the first experiment) as described above. We also included the six symbiont‐free lines in the experiment. For each line, we exposed 40 aphids to *Pandora* and kept 40 aphids as a control. Data were analyzed using GLMs as above. Host genotype was nested within host biotype, and modeled as a fixed effect. As above, there were no block effects to include in the model.

Last, we assessed the costs of *Regiella* infection by analyzing the survival of control aphids as a proxy for host fitness. A survival analysis was performed using a Cox Proportional Hazards model, with *Regiella* presence or absence and *Regiella* genotype as fixed effects. A test of the proportional hazards assumption was conducted to ensure the data fit model assumptions. A survival coefficient was calculated for each symbiont genotype, and we used a Spearman's rank correlation test to compare the rank‐order of model coefficients for survival and symbiont‐conferred protection, using the survival package in R v. 3.0.2 (Therneau and Grambsch 2000).

## Results

### SPECIFICITY OF SYMBIONT BY PARASITE GENOTYPES (G_Symbiont_ x G_Parasite_)

**Figure 2:**
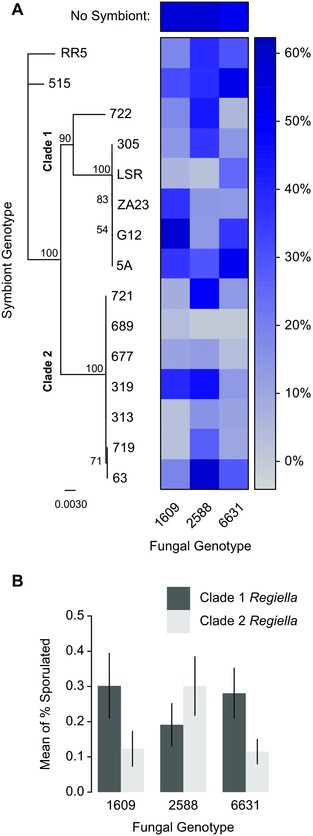
G_Symbiont_ x G_Parasite_ interactions. (A) *Regiella* genotype and phylogenetic structure are shown to the left of A. The top clade (referred to here and elsewhere as “clade 1”) is associated with the *Medicago sativa* aphid biotype; the bottom clade (“clade 2”) is associated with the *Trifolium* spp. aphid biotype. The heat‐map shows the percentage of aphids in each combination of symbiont and pathogen genotype that produced a sporulating cadaver. Aphids without *Regiella* are shown at the top of the figure. Darker blue boxes represent a higher rate of sporulation, indicating weaker protection by *Regiella* against a *Pandora* genotype. Pathogen genotype is indicated at the bottom of the figure. (B) Figure 2B shows these same data with the main *Regiella* clades grouped together into dark and light bars. Fungal genotype is indicated at the bottom of the figure; the *y*‐axis shows the mean of the sporulation rates of each symbiont clade. Error bars show standard error.

We were able to successfully establish 15 aphid lines each with a different *Regiella* genotype in a common host genetic background. We then exposed these lines to three genotypes of *Pandora*. Fungal genotype had a significant but small effect on the percentage of aphids that produced a sporulating cadaver (explaining 2.4% of the deviance accounted for by a model including all factors and their interaction; Table 1, Fig. 2A). The presence of *Regiella* significantly influenced sporulation rate, explaining 25.8% of the deviance (Table 1, Fig. 2A). Symbiont genotype explained a further 39.0% of the deviance for 14 degrees of freedom (Table 1), and approximately one fifth of this was due to differences between the two main phylogenetic clades of *Regiella* harbored by pea aphids (Henry et al. 2013) (Fig. 2A). This shows that *Regiella* genotypes differ in how well they protect hosts against the *Pandora* genotypes used in this study.

**Table 1:** Results of analysis of deviance for generalized linear models

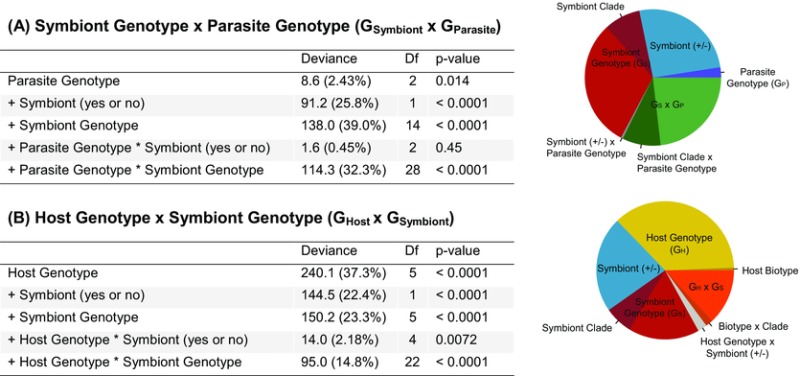

We investigated the effects of symbiont and parasite genotypes (**A**: G_Symbiont_ x G_Parasite_ experiment) and host and symbiont genotypes (**B**: G_Host_ x G_Symbiont_ experiment) on the percent of aphids that produce a sporulating cadaver. The pie graphs to the right of the table indicate the percent of the difference in deviance between the minimal and full models that is explained by each factor. The proportion of each factor explained by symbiont clade and/or aphid biotype, when applicable, is shown with a darker color.

The interaction between *Regiella* presence or absence and fungal genotype was not significant, indicating that the *Pandora* genotypes we used were equally susceptible to *Regiella* defenses. However, there was a significant interaction between *Pandora* genotype and *Regiella* genotype (explaining 32.3% of deviance; Table 1, Fig. 2A), showing that genetic interactions between pathogens and protective symbionts influence infection rate. Of this variance, approximately one‐third was explained by the interaction between *Pandora* genotype and *Regiella* clade. The two main *Regiella* clades therefore differ in terms of how strongly they protect hosts against specific *Pandora* genotypes (Fig. 2B), implying that pathogen genotypes are to some extent adapted to overcome the defenses of symbionts from specific clades, and vice versa.

### HOST BY SYMBIONT GENOTYPIC INTERACTIONS (G_Host_ x G_Symbiont_)

**Figure 3:**
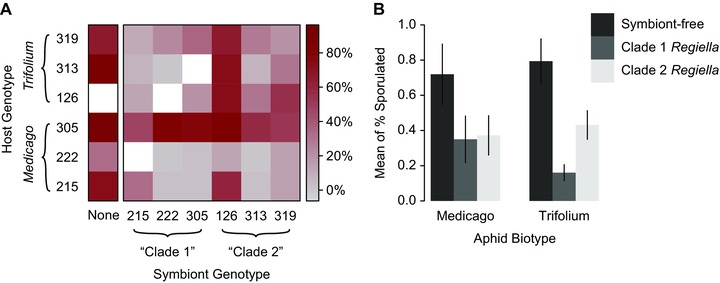
G_Host_ x G_Symbiont_ interactions. (A) Darker red boxes represent a higher rate of sporulation, indicating weaker protection by a host‐symbiont pair against *Pandora*. Symbiont genotype is indicated along the bottom of the figure, and host genotype is indicated along the left side. Symbiont‐free aphids from each of the six host genotypes are show in the left‐most column. Original, native host‐symbiont pairs are shown along a diagonal. Aphid genotypes are grouped into *Trifolium* and *Medicago* biotypes, as indicated. (B) Figure 3B shows the same results with the *Medicago* and *Trifolium* biotypes grouped together along the *x*‐axis, and symbiont‐free aphids, aphids harboring clade 1 *Regiella*, and aphids harboring clade 2 *Regiella* grouped into dark, medium, and light bars, respectively. The *y*‐axis shows mean sporulation, and error bars show standard error.

We generated a set of lines involving six aphid and six *Regiella* genotypes to test whether G_Host_ x G_Symbiont_ specificity influences resistance against a single *Pandora* genotype. We were unable to establish three combinations of hosts and symbionts prior to the experiment, and one line died out while we were rearing the aphids for use in the experiment (Fig. 3A). We were subsequently able to establish the three missing host plus symbiont combinations after the experiment, demonstrating that these were not incompatible genotypes.

Host genotypes differed in their susceptibility to *Pandora* and this factor accounted for 37.3% of the deviance explained by the full model we fitted (Table 1; Fig. 3A). Host biotype, however, explained less than 2% of this figure. As in our first experiment, the presence or absence of *Regiella* in a host line significantly influenced sporulation rate (explaining 22.4% of deviance, Table 1). We again found significant variation among *Regiella* genotypes (Table 1) accounting for 23.3% of deviance, of which one third was explained by *Regiella* clade. We found an interaction between *Regiella* and host genotype responsible for 14.8% of deviance (Table 1), a significant G_Host_ x G_Symbiont_ effect. Approximately one‐tenth of this value was explained by the interaction between host biotype and symbiont clade, and we found protection was not stronger in each symbiont's native host genetic background (Fig. 3B). This implies that in terms of the strength of protection conferred, *Regiella* clades are not strongly adapted to specific host biotypes. Lastly, we found a small effect of the interaction between host genotype and the presence or absence of a symbiont (explaining 2.18% of deviance, Table 1), indicating that host genotypes varied slightly in how well they were protected by *Regiella*, irrespective of symbiont genotype.

### COSTS OF SYMBIONT‐CONFERRED PROTECTION

We used the data from our panel of 15 *Regiella* genotypes in a common host background to address whether there is an association between the strength of symbiont‐conferred protection and the costs of carrying the symbiont for the host. Harboring *Regiella* reduced host survival (data from control aphids not exposed to pathogen: χ^2^ = 9.01, 1 d.f., *P* = 0.0027), and the genotype of *Regiella* influenced the magnitude of this cost (χ^2^ = 96.4, 15 d.f., *P* <0.0001; Fig. S3). We tested for a correlation between the residuals of this regression and the residuals of the G_Symbiont_ x G_Parasite_ analysis described above, using symbiont genotype as the unit of replication. We found no association between the two traits (Spearman rank correlation test: S = 632, *P* = 0.65; Fig. S3). The cost of carrying a symbiont is therefore not correlated with the strength of protection the symbiont confers in this system.

## Discussion and Conclusions

The microbial symbionts of eukaryotes often show substantial genotypic variation, with consequences for the phenotypic effects they have on their carriers, including resistance against natural enemies (Scheublin et al. 2007; Duron and Hurst 2013; Martinez et al. 2014, 2015; McLean and Godfray 2015). An important question in symbiont biology is how natural selection maintains this variation (Heath and Stinchcombe 2014). Host genetic variation in natural enemy resistance has long been known and is thought to be maintained (1) by the activation and maintenance costs of immunological responses coupled with spatio‐temporal variation in the probability of attack (Sheldon and Verhulst 1996; Schulenburg et al. 2009), and (2) through specific interactions between host and natural enemy genotypes giving rise to negative‐frequency dependent selection (Lively and Dybdahl 2000). We measured the fitness costs to aphids of harboring *Regiella*, but found no association between the relative costs of harboring different symbiont genotypes and the strength of protection they conferred against *Pandora*, though we note that the costs of symbiont‐mediated protection could be manifest in ways that we did not measure here. This is in contrast with what has been found in other systems, such as the *Wolbachia* bacteria harbored by *Drosophila simulans* that provide protection against RNA viruses, where the strength of protection found among symbiont genotypes is associated with costs to the host (Martinez et al. 2015). We instead found that resistance was influenced by the interaction of symbiont and fungal genotypes (G_Symbiont_ x G_Parasite_). This suggests that strain diversity among protective symbionts may be maintained by parasite genetic diversity, potentially subject to the type of coevolutionary dynamics observed involving host and parasite genotypes (Kwiatkowski et al. 2012; Heath and Stinchcombe 2014). In addition, symbiont strains differed in the effectiveness of protection they provided independently of *Pandora* genotype. If providing benefits to hosts is costly for symbionts, perhaps in terms of transmission efficiency or competition between co‐infecting symbiont strains or species (Ferrari and Vavre 2011), this finding could indicate the potential for symbionts to lose their protective function and become low‐quality partners (Jones et al. 2015). Indeed, a recent study of pea aphid symbionts under natural conditions demonstrated that while symbionts do confer protection against natural enemies in the field, the net benefit of harboring a symbiont might be close to zero or even negative for hosts, and any benefits depend on ecological context (including the communities of natural enemies present in a population) (Hrček et al. 2016). Future work should consider host‐endosymbiont associations within broader ecological communities (McLean et al. 2016), and within the context of the mutualism‐parasitism spectrum (Bronstein 1994).

In addition to G_Symbiont_ x G_Parasite_ interactions, we also found that symbiont‐mediated protection is influenced by the interaction between host and symbiont genotypes. One potential explanation for this pattern would be the reciprocal adaptation of co‐occurring host and symbiont lineages (Parker 1999). The *Regiella* found in pea aphids form two major phylogenetic clades (Henry et al. 2013), which are associated with particular biotypes feeding on the host plants *Medicago sativa* and *Trifolium* spp. (Ferrari et al. 2012). However, symbionts in their “natural” host genetic background did not confer stronger pathogen protection than in other hosts. In addition, the observed G_Host_ x G_Symbiont_ effects appeared idiosyncratic and were not explained by major symbiont clade or host biotype. Our data therefore do not support a model of reciprocal adaptation between lineages leading to mutually beneficial effects of the symbiont on the host. These findings are perhaps surprising given the predominantly vertical transmission of *Regiella*—vertical transmission tends to favor the evolution of mutualism, as microbes have a strong evolutionary interest in the fitness of their hosts (Herre et al. 1999). The expectation is that the rapid genetic dynamics that are characteristic of more antagonistic interactions will be absent from beneficial interactions (Law and Lewis 1983; Law and Koptur 1986; Weyl et al. 2010). However, some researchers have suggested that the evolutionary interests of hosts and vertically transmitted symbionts may not be as fully aligned as previously thought, allowing for the spread and persistence of mutations that favor the symbiont at the expense of the host even in associations where the partners show high mutual dependence (Frank 1997; Douglas and Werren 2016). An exciting possibility discussed in the recent literature is that elements of Red Queen dynamics may occur in these associations (Bennett and Moran 2015). So far there has been little evidence to test this idea (though see Heath and Tiffin (2007) for an example of G x G interactions between plants and horizontally acquired Rhizobial fungi). We found a significant G_Host_ x G_Symbiont_ interaction, little evidence of adaptation between co‐occurring lineages, and evidence of weakly protective or “cheater” strains. Antagonistic coevolution between hosts and predominantly vertically transmitted symbionts is one potential explanation for these patterns, but more data are needed.

G_Host_ x G_Symbiont_ interactions might also influence the success of novel pairings of host and symbiont genotypes, which occur either through horizontal transmission events or after sexual reproduction when vertically transmitted symbionts will encounter novel host backgrounds produced by host genetic recombination. Some studies of horizontal transfer of symbionts among hosts have found symbionts to retain their function in new host backgrounds both after movement between (Russell and Moran 2006; Łukasik et al. 2015) and within species (Hansen et al. 2012; Haselkorn and Jaenike 2015). Others have found that the phenotypic effects of symbionts vary in different host species, indicating a potential for host genotype to influence horizontal transmission (Tinsley and Majerus 2007; Russell et al. 2009; Haselkorn and Jaenike 2015). Symbionts have been described as constituting a “horizontal gene pool” of useful adaptations (akin to the pool of mobile genetic elements available to microorganisms), from which eukaryotes can rapidly acquire novel phenotypes and therefore gain competitive advantages (Moran 2007; Jaenike et al. 2010; Jaenike 2012). Our results suggest that interactions between host and symbiont genotypes influence the fitness effects of harboring symbionts for hosts, and therefore have an important influence on the capacity for adaptation through symbiosis.

We have demonstrated that the strength of symbiont‐mediated resistance is dependent on genotype by genotype specificity between symbionts and both hosts and parasites. Reciprocal evolutionary change between interacting species, or coevolution, can only occur if genotype by genotype interactions affect fitness‐related traits (Heath and Nuismer 2014). Our study therefore represents a significant step in understanding how protective symbionts can influence host‐parasite coevolution. Parasites have to overcome both host immunological and symbiont‐based defenses (Parker et al. 2011), and we have shown that they also have to adapt to strain‐specific features of protective microbes. In turn, host immune systems manage the dual role of combating variable parasite genotypes while fostering and policing interactions with potentially beneficial microbes. We have identified an additional adaptive challenge for hosts: interacting with variable symbiont genotypes that differ in their phenotypic effects. Genetic variation among hosts, parasites, and protective symbionts will therefore reflect complex interactions among all three players, and these interactions are expected to play important roles in coevolution.

Associate Editor: A.‐L. Laine

Handling Editor: P. Tiffin

## Supporting information


**Figure S1**. G_S_ x G_P_ data, shown as a bar graph, with percent sporulation on the *y*‐axis.
**Figure S2**. G_H_ x G_S_ data, shown as a bar graph, with percent sporulation on the *y*‐axis.
**Figure S3**. Correlation between symbiont‐mediated protection and the costs of harboring a symbiont across *Regiella* genotypes.
**Table S1**. Information on the aphid genotypes used.
**Table S2**. Information on *Regiella* symbiont genotypes used.
**Table S3**. Information on fungal pathogen genotypes used.
