# Supplementary material for: Genotype specificity among hosts, pathogens, and beneficial microbes influences the strength of symbiont‐mediated protection
Source: Evolution. 2017 Mar 24;71(5):1222–31. doi: 10.1111/evo.13216 (PMC5516205; doi:10.1111/evo.13216)
Supplement: Supplementary file 1 — Figure S1. GS x GP data, shown as a bar graph, with percent sporulation on the y‐axis. Figure S2. GH x GS data, shown as a bar graph, with percent sporulation on the y‐axis. Figure S3. Correlation between symbiont‐mediated protection and the costs of harboring a symbiont across Regiella genotypes. Table S1. Information on the aphid genotypes used. Table S2. Information on Regiella symbiont genotypes used. Table S3. Information on fungal pathogen genotypes used. [file EVO-71-1222-s001.pdf]

Supplementary Information

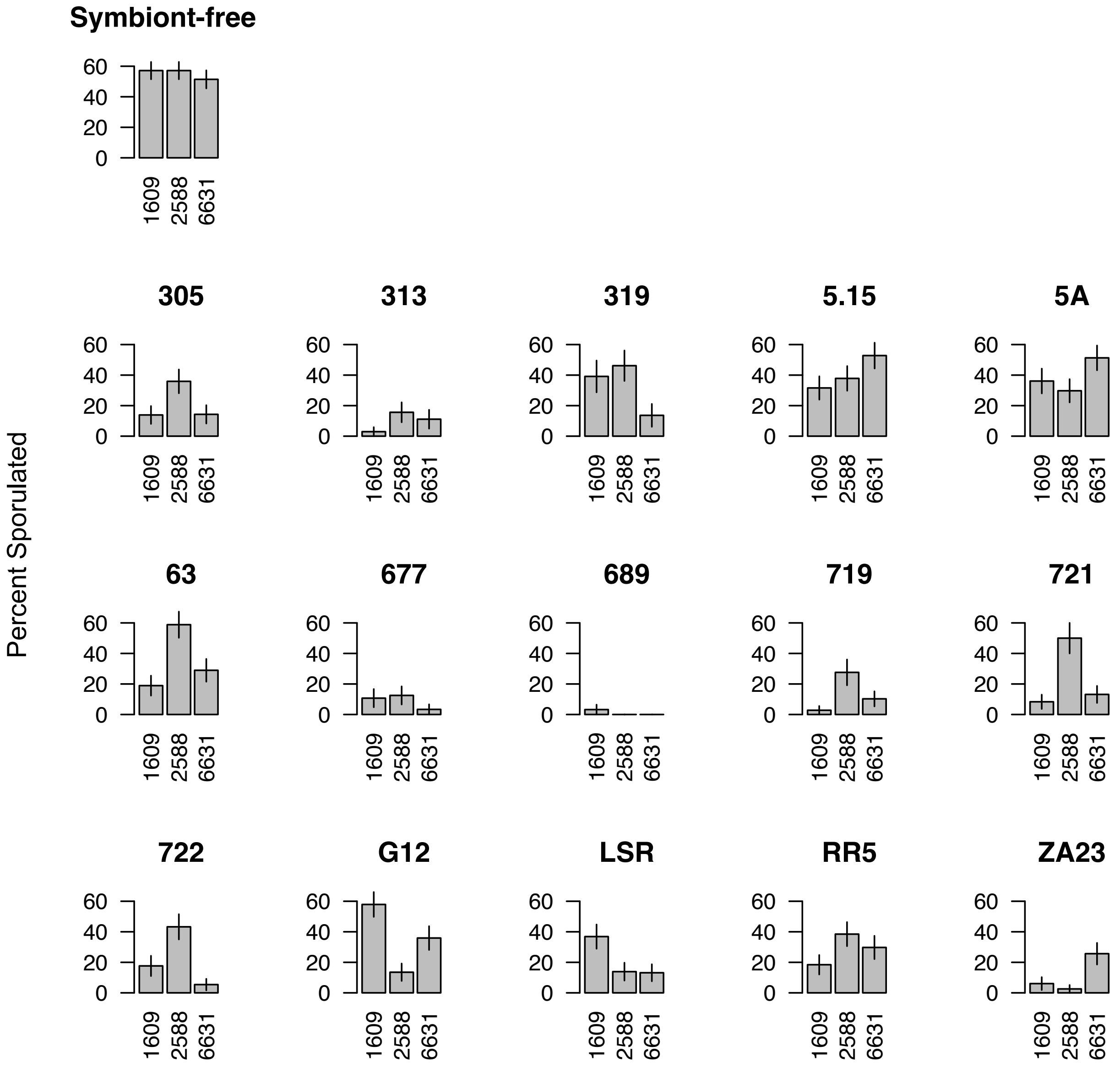

Supplementary Figure 1: G_S x G_P data, shown as a bar graph, with percent sporulation on the y-axis. Each symbiont genotype is shown as a separate graph, with fungal genotypes along the x-axis. This is the same data shown in Figure 2 in the main text. Error bars show standard error.

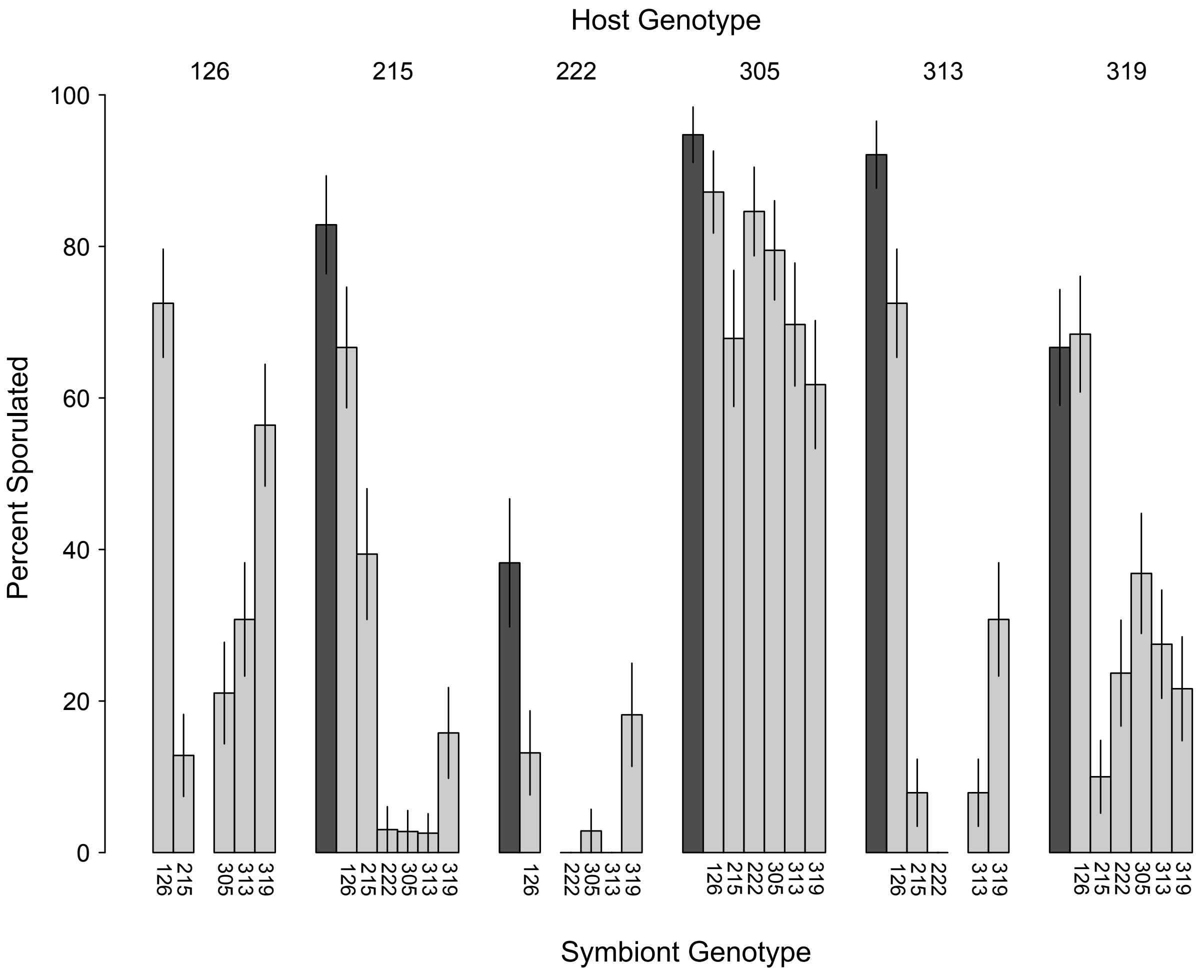

Supplementary Figure 2: G_H x G_S data, shown as a bar graph, with percent sporulation on the y-axis. The aphid genotypes are listed at the top of the graph, and symbiont genotypes are indicated at the bottom. This is the same data shown in Figure 3 in the main text. Dark bars represent aphids from each genotype without *Regiella*. Error bars show standard error.

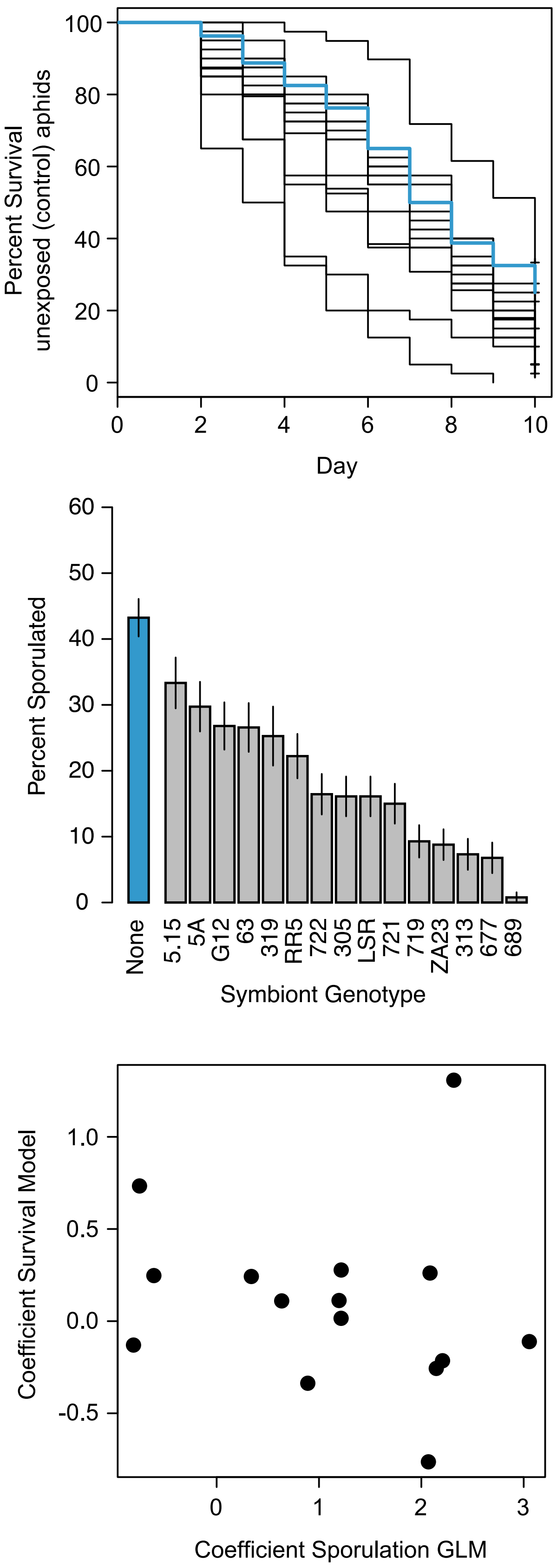

Supplementary Figure 3: Correlation between symbiont-mediated protection and the costs of harboring a symbiont across *Regiella* genotypes. **Top:** A graph of control aphid survival for the G_S x G_P experiment. Each black line shows the survival of unexposed aphids during the course of the experiment, with each line representing a different *Regiella* genotype. The blue line shows aphids without *Regiella*. **Middle**: Percent sporulation of each *Regiella* genotype, averaged across the 3 *Pandora* genotypes. Despite the importance of symbiont genotype by pathogen genotype interactions in determining infection rate, symbiont genotypes differed independently from pathogen genotype in how well they protected hosts from *Pandora*, shown here. Error bars show standard error. The blue bar shows aphids without *Regiella*. **Bottom:** Correlation between symbiont-mediated protection and the costs of harboring *Regiella*. The coefficients from two statistical analyses, a GLM of percent sporulation (where a higher number indicates more sporulation, indicating weaker protection), and a survival analysis of control aphids (a higher number indicates lower survival, indicating stronger costs), are shown here. There was no significant correlation between these two traits across the *Regiella* genotypes.

Supplementary Table 1: Information on the aphid genotypes used.

Host Genotype	Location Collected	Year Collected	Original Symbionts	Used in which experiment
126	Legoland, UK	2003	*Regiella*	H*S
215	Lincoln, UK	2012	*Regiella*	H*S
222	Whitby Field, UK	2003	*Regiella, Hamiltonella, Rickettsia*	H*S
305	Wharburg Reserve, UK	2003	*Regiella, Hamiltonella*	H*S
313	Upper Slaughter, UK	2007	*Regiella*	H*S
319	Oddington, UK	2012	*Regiella*	H*S
145	Windsor Ranger's Gate, UK	2003	None	S*P

Supplementary Table 2: Information on *Regiella* symbiont genotypes used.

Symbiont Genotype	Location Collected	Year Collected	Co-infecting symbionts (before isolation)	Aphid species collected	Used in which experiment
RR5	Rothamsted Research Center, UK		None	*Sitobion avenae*	S*P
515	Bacchus Marsh, UK	2003	None	*Myzus persicae*	S*P
722	DOM, France	2014	None	*Acyrthosiphon pisum*	S*P
305	Wharburg Reserve, UK	2003	*Hamiltonella*	*Acyrthosiphon pisum*	S*P & H*S
ZA23	Milledgeville PA, USA	2010	None	*Acyrthosiphon pisum*	S*P
LSR	Ithaca, NY, USA	1998	None	*Acyrthosiphon pisum*	S*P
G12	Atlanta, GA, USA	2008	None	*Acyrthosiphon pisum*	S*P
5A	Tompkins County, NY, USA	2000	None	*Acyrthosiphon pisum*	S*P
721	DOM, France	2014	None	*Acyrthosiphon pisum*	S*P
689	Milford on sea, UK	2014	None	*Acyrthosiphon pisum*	S*P
677	“Crawley’s Roundabout”, Berkshire, UK	2014	None	*Acyrthosiphon pisum*	S*P
319	Oddington, UK	2012	None	*Acyrthosiphon pisum*	S*P & H*S
313	Upper Slaughter, UK	2007	None	*Acyrthosiphon pisum*	S*P & H*S
719	LR, France	2014	None	*Acyrthosiphon pisum*	S*P
63	Windsor Ranger's Gate, UK	2010	None	*Acyrthosiphon pisum*	S*P
222	Whitby Field, UK	2003	*Hamiltonella, Rickettsia*	*Acyrthosiphon pisum*	H*S
215	Lincoln, UK	2012	None	*Acyrthosiphon pisum*	H*S
126	Legoland, UK	2003	None	*Acyrthosiphon pisum*	H*S

Supplementary Table 3: Information on fungal pathogen genotypes used.

**Pandora Genotype**	**Aphid species collected**	**Location (host plant)**	**Date**
2588	Acyrthosiphon pisum	Lansing, New York, USA (alfalfa)	20 July 1988
1609	Acyrthosiphon pisum	La Miniere, France	19 September 1984
6631	Acyrthosiphon pisum	Latah County, Idaho, USA (garden pea)	25 June 2000
